# The Genetics of Non-conventional Wine Yeasts: Current Knowledge and Future Challenges

**DOI:** 10.3389/fmicb.2015.01563

**Published:** 2016-01-11

**Authors:** Isabelle Masneuf-Pomarede, Marina Bely, Philippe Marullo, Warren Albertin

**Affiliations:** ^1^ISVV, Unité de Recherche Œnologie EA 4577, USC 1366 Institut National de la Recherche Agronomique, Bordeaux INP, University BordeauxVillenave d'Ornon, France; ^2^Bordeaux Sciences AgroGradignan, France; ^3^BiolaffortBordeaux, France; ^4^ENSCBP, Bordeaux INPPessac, France

**Keywords:** non-conventional yeast, non-Saccharomyces, wine, enology, oenology, microsatellite

## Abstract

*Saccharomyces cerevisiae* is by far the most widely used yeast in oenology. However, during the last decade, several other yeasts species has been purposed for winemaking as they could positively impact wine quality. Some of these non-conventional yeasts (*Torulaspora delbrueckii, Metschnikowia pulcherrima, Pichia kluyveri, Lachancea thermotolerans*, etc.) are now proposed as starters culture for winemakers in mixed fermentation with *S. cerevisiae*, and several others are the subject of various studies (*Hanseniaspora uvarum, Starmerella bacillaris*, etc.). Along with their biotechnological use, the knowledge of these non-conventional yeasts greatly increased these last 10 years. The aim of this review is to describe the last updates and the current state-of-art of the genetics of non-conventional yeasts (including *S. uvarum, T. delbrueckii, S. bacillaris*, etc.). We describe how genomics and genetics tools provide new data into the population structure and biodiversity of non-conventional yeasts in winemaking environments. Future challenges will lie on the development of selection programs and/or genetic improvement of these non-conventional species. We discuss how genetics, genomics and the advances in next-generation sequencing will help the wine industry to develop the biotechnological use of non-conventional yeasts to improve the quality and differentiation of wines.

## Introduction

In oenology, alcoholic fermentation is generally performed by *Saccharomyces cerevisiae* yeast, the “conventional” wine yeast. Currently, the winemakers have the choice between hundreds of *S. cerevisiae* starters that have been selected for various characteristics including their ability to complete alcoholic fermentation in oenological conditions, their low release of off-flavor compounds, their positive impact on wine aromas, etc., (Pretorius, 2000; Marullo and Dubourdieu, 2010). The growing demand for more diversified wines or for specific characteristics (low ethanol content, etc.) has led to the exploration of new species for winemaking. These non-conventional yeasts may contribute to the wine's flavor and taste by producing a broad range of secondary metabolites and extracellular enzymes (Hong and Park, 2013; Ciani et al., 2014; Wang et al., 2015). Some species could be interesting for alcohol level reduction in wine (Masneuf-Pomarede et al., 2010; Bely et al., 2013) or for greater fermentative ability in harsh conditions due to enhanced fructophily (Sutterlin, 2010; Magyar and Tóth, 2011). It has to be noted that, as only some *Saccharomyces* species (i.e., *S. cerevisiae, S. uvarum*, and some interspecific hybrids) are able to consume all the sugar contained in grape must, non-*Saccharomyces* yeasts must be used in co- or sequential-fermentation with a *Saccharomyces* spp. able to secure AF completion (Jolly et al., 2006; Bely et al., 2013).

**Figure 1:**
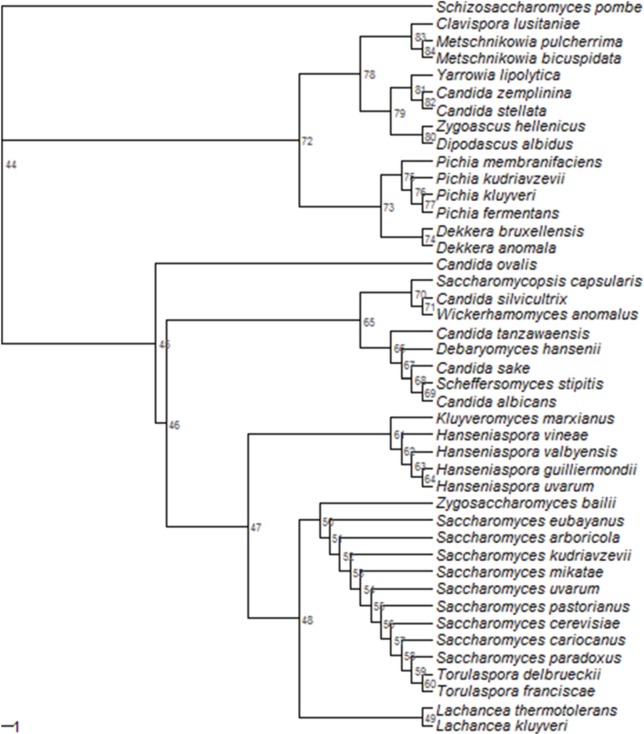
**Phylogeny of 41 species of Saccharomycetales on the basis of 18S ribosomal DNA sequence**. Multiple sequence alignment (1951 bases) was performed by Clustal Omega (EMBL-EBI website). Genetic distance was computed using the K80 Kimura model (Kimura, 1980), phylogenetic tree was built using Neighbor joining clustering method and bootstrapping (1000 replicates) was used to assess the robustness of the nodes by means of R package ape (Paradis et al., 2004). *Schizosaccharomyces pombe* was used as outgroup species. The following sequences and strains (mostly type strains) were used: AB000642.1|*Dipodascus albidus* IFO 1984; AB013504.1|*C. tanzawaensis* JCM 1648; AB018175.1|*C. stellata* JCM 9476; AB023473.1|*M. pulcherrima* IFO 1678; AB040997.1|*S. kudriavzevii* IFO 1802; AB040998.1|*S. mikatae* IFO 1815; AB054561.1|*C. silvicultrix* JCM 9831; AB013529.1|C. sake JCM 2951; AF548094.1|*S. cerevisiae* CBS 1171; AJ271813.1|*S. cariocanus* UFRJ 50816; AY046254.1|*H. valbyensis* NRRL Y-1626; AY046256.1|*H. guilliermondii* NRRL Y-1625; AY046257.1|*H. uvarum* NRRL Y-1614; AY046258.1|*H. vineae* NRRL Y-17529; *S. bacillaris* CBS 9494; EF550365.1|*P. membranifaciens* NRRL Y-2026; EF550372.1|*P. fermentans* Y-1619; EF550389.1|*P. kluyveri* NRRL Y-11519; EF550396.1|*D. anomala* NRRL Y-17522; EF550479.1|*Wickerhamomyces anomalus* NRRL Y-366; EU011714.1|*C. ovalis* NRRL Y-17662; EU011734.1|*D. bruxellensis* NRRL Y-12961; EU348783.1|*C. albicans* NRRL Y-12983; FJ153136.1|*L. thermotolerans* NRRL Y-8284; FJ153143.1|*T. franciscae* NRRL Y-6686; GU266277.1|*S. arboricola* AS 2.3317; GU597328.1|*Zygoascus hellenicus* CBS 5839; HQ651939.1|*Scheffersomyces stipitis* ATCC 58376; JQ698884.1|*Saccharomycopsis capsularis* NRRL Y-17639; JQ698900.1|*Clavispora lusitaniae* NRRL Y-11827; JQ698910.1|*Debaryomyces hansenii* NRRL Y-7426; JQ698926.1|*Yarrowia lipolytica* NRRL YB-423; JQ698936.1|*Schizosaccharomyces pombe* NRRL Y-12796; M55528.1|*P. kudriavzevii* MUCL 29849; *S. eubayanus* FM1318; *S. uvarum* CBS7001; X69846.1|*M. bicuspidata* MUCL 31145; X89523.1|*L. marxianus* CBS 712; X91083.1|*Zygosaccharomyces bailii* NCYC 1416; X97805.1|*S. pastorianus* NCYC 392; X97806.1|*S. paradoxus* CBS 432; X98120.1|*T. delbrueckii* CBS 1146; Z75580.1|*L. kluyveri* NCYC 543.

The wine industry currently proposes starters of a few non-conventional yeasts (*Torulaspora delbrueckii, Metschnikowia pulcherrima, Pichia kluyveri, Lachancea thermotolerans*, etc.), while several other species (*Hanseniaspora uvarum, Starmerella bacillaris*, etc.) are the subject of various studies to assess both positive contribution (Table 1) and negative impact (if any) on wine quality (Bely et al., 2013; Maturano et al., 2015). These non-conventional yeasts are widely distributed amongst the *Saccharomycetales* (Figure 1). In order to evaluate the oenological potential of a given species, several strains are usually compared for phenotypes of interest like fermentation ability (Renault et al., 2009) or glycerol production (Magyar and Tóth, 2011). However, in most cases, neither the relationships between the tested strains are described, nor the genetic structuration of the species is known. This lack of genetic knowledge is clearly detrimental, since we are not able to determine whether the phenotypic diversity described is representative of the species or not. The recent advances in next-generation sequencing (NGS) have triggered the development of genomic and genetic tools for some of these non-conventional yeasts, but the field is still in its infancy. The objective of this paper is thus to review the current state-of-art of the genetics of non-conventional wine yeasts and to discuss the future prospects and challenges from an oenological viewpoint.

**Table 1:** **Comparison of wine yeast species**.

**Species/synonym (anamorph)**	**Features of interest in winemaking**	**Genome size**	**Full nuclear genome sequence**	**Basic ploidy level**	**Sporulation/zygote formation**	**Heterozygosity^a^**	**Ecological niches**	**Genetic subgroups**	**Genetic diversity from winemaking environments^b^**
*Saccharomyces cerevisiae*	AF completion	Nucleus: 12.0 Mb, 16 chromosomes (Goffeau et al., 1996). Mitochondrion: 85 Kb (Foury et al., 1998).	Several hundred sequences: lab strain S288c (Goffeau et al., 1996), wine strains EC1118 (Novo et al., 2009) and AWRI1631 (Borneman et al., 2008), the 100-genomes strains (Strope et al., 2015), etc.	Diploid, occasional tetraploid associated with specific environments (Albertin et al., 2009; Al Safadi et al., 2010)	4 spores per ascus. Zygotes readily observed. (Kurtzman et al., 2011)	75.1–81.9% (308/410 clones, 136/166 clones) (Legras et al., 2007; Muller and McCusker, 2009)	Wild environments: fruit, plant, insect, soil. Anthropic environments: wine, other distilled and traditional fermented beverages, food fermentation, dairy product, bioethanol. Lab environments. Clinical environments. (Fay and Benavides, 2005; Legras et al., 2007; Kvitek et al., 2008; Diezmann and Dietrich, 2009; Schacherer et al., 2009; Wang et al., 2012)	Wild and domestic populations associated with wine, beer, bread, etc. (Fay and Benavides, 2005; Legras et al., 2007; Almeida et al., 2015), multiple domestication events (Schacherer et al., 2009).	0.39–0.65 (Albertin et al., 2014b); 0.00–1.00 (Schuller et al., 2012); 0.27–0.35 (Hall et al., 2011)
*Saccharomyces uvarum*	AF completion (Masneuf-Pomarede et al., 2010); reduced ethanol production (Bely et al., 2013); psychrophilism (Masneuf-Pomarede et al., 2010); Acetate ester production (Masneuf-Pomarede et al., 2010)	Nucleus: 11.5 Mb, 16 chromosomes (Almeida et al., 2014).	More than 50 genomes of which CBS7001^T^ (Cliften et al., 2003; Almeida et al., 2014)	Diploid	4 spores per ascus. Zygotes readily observed. (Kurtzman et al., 2011)	0% (0/40 strains) (Masneuf-Pomarede et al., 2007)	Wild environments: plant. Anthropic environments: wine, cider. (Almeida et al., 2014)	Wild and domestic populations associated with wine and cider (Almeida et al., 2014)	0.00–0.62 (Masneuf-Pomarede et al., 2007)
*Torulaspora delbrueckii* (*Candida colliculosa*)	Volatile acidity reduction (Bely et al., 2008); Aroma and complexity (Ciani and Maccarelli, 1998; Renault et al., 2009; Azzolini et al., 2012)	Nucleus: 9.2–11.5 Mb, 8 chromosomes (Gordon et al., 2011; Gomez-Angulo et al., 2015). Mitochondrion: 28–45 Kb (Wu et al., 2015).	2 genomes: CBS 1146^T^ and NRRL Y-50541 (Gordon et al., 2011; Gomez-Angulo et al., 2015)	Unclear, could be diploid (Albertin et al., 2014a)	One spore per ascus, occasional 2–3 spores/ascus (Kurtzman et al., 2011; Albertin et al., 2014a).	26.4% (29/110 strains) (Albertin et al., 2014a)	Wild environments: fruit, plant, insect, soil. Anthropic environments: wine, other distilled and traditional fermented beverages, food fermentations, dairy products. (Albertin et al., 2014a)	Wild and domestic populations associated with wine and other bioprocesses, geographical clustering for wild populations (Albertin et al., 2014a).	0.35–1.00 (Albertin et al., 2015)
*Hanseniaspora uvarum* (*Kloeckera apiculate*)	Aroma (Rojas et al., 2001)	Nucleus: 8.08–9.08 Mb, 8 to 9 chromosomes (Esteve-Zarzoso et al., 2001). Mitochondrion: 11Kb (Pramateftaki et al., 2006).	2 genomes: DSM 2768 and 34–9 (NCBI^1^)	Unclear, could be diploid (Albertin et al., 2016)	One, seldom two spores per ascus (Kreger-van Rij, 1977). Zygotes described^3^.	82.6% (95/115 strains) (Albertin et al., 2016)	Wild environments: fruit, plant, insect, bird, mollusc, shrimp, soil. Anthropic environments: wine, other distilled and traditional fermented beverages. (Grangeteau et al., 2015; Albertin et al., 2016)	Geographical and temporal clustering (Albertin et al., 2016).	1.00 (but low number of strains per sample) (Albertin et al., 2016).
*Hanseniaspora guillermondii* (*Kloeckera apis*)	Acetate ester production (Rojas et al., 2001; Moreira et al., 2008; Viana et al., 2008)	Nucleus: 8 to 9 chromosomes (Esteve-Zarzoso et al., 2001).	–	–	Four spores per ascus (Barnett et al., 2000). Zygotes described^3^.	–	Wild environments: fruit, soil. Anthropic environments: wine.	–	–
*Hanseniaspora vinae* (*Kloeckera africana*)	Acetate ester production (Viana et al., 2011)	Nucleus: 11.4 Mb, 5 chromosomes (Esteve-Zarzoso et al., 2001; Giorello et al., 2014).	1 genome: T02/19AF (Giorello et al., 2014)	–	One, seldom two spores per ascus (Kreger-van Rij, 1977).	–	Anthropic environments: wine.	–	–
*Starmerella bacillaris* (*Candida zemplinina*)	Fructophily (Magyar and Tóth, 2011; Tofalo et al., 2012; Englezos et al., 2015); reduced ethanol production (Di Maio et al., 2012; Bely et al., 2013; Giaramida et al., 2013); glycerol production (Di Maio et al., 2012; Giaramida et al., 2013; Zara et al., 2014); Aroma release (Andorrà et al., 2012); other characteristics (Mangani et al., 2011; Sadoudi et al., 2012; Tofalo et al., 2012; Domizio et al., 2014; Magyar et al., 2014)	Nucleus: 3 chromosomes (Sipiczki, 2004). Mitochondrion: 23 Kb (Pramateftaki et al., 2008).	–	Unclear, could be haploid (Masneuf-Pomarede et al., 2015)	No evidence of sporulation ability (Masneuf-Pomarede et al., 2015)	0.01% (1/163) (Masneuf-Pomarede et al., 2015)	Rare in wild environments. Anthropic environments: grape and wine. (Masneuf-Pomarede et al., 2015)	No evidence of domestication event, geographical clustering. (Masneuf-Pomarede et al., 2015)	0.90–0.97 (Masneuf-Pomarede et al., 2015)
*Candida stellata*/*Torulopsis stellata*	Glycerol production (Ciani and Maccarelli, 1998); Fructophily (Magyar and Tóth, 2011)	Nucleus: 3 chromosomes (Sipiczki, 2004)	–	–	No evidence of sporulation ability	–	Anthropic environments:wine (Csoma and Sipiczki, 2008)	–	–
*Lachancea thermotolerans* /*Kluyveromyces thermotolerans*	Glycerol overproduction (Comitini et al., 2011); Acetate ester production (Comitini et al., 2011); reduction of volatile acidity (Comitini et al., 2011)	Nucleus: 10.4 Mb, 8 chromosomes (Malpertuy et al., 2000). Mitochondrion: 21.9–25.1 Kb (Talla et al., 2005; Freel et al., 2014).	1 genome: CBS 6340^T^ (Malpertuy et al., 2000)	Controversial: haploid (Freel et al., 2014) or diploid (Souciet et al., 2009)	One to four spores per ascus (Barnett et al., 2000). Zygotes described^3^.	–	Wild environments: fruit, plant. Anthropic environments:wine and agave fermentations (Freel et al., 2014)	Geographical clustering (Freel et al., 2014)	–
*Lachancea kluyveri*	NA	Nucleus: 11.3 Mb, 8 chromosomes (Souciet et al., 2009). Mitochondrion: 49–53.7 Kb piskur 1998; 51.5 (Jung et al., 2012)	1 genome: NCYC 543^T^ (Souciet et al., 2009)	Diploid, occasional triploid (Freel et al., 2014)	–	–	Wild environments: soil, insect, plant (Jung et al., 2012).	Geographical clustering (Jung et al., 2012)	–
*Debaryomyces hansenii/Pichia hansenii (Candida famata*)	Enzymatic activities (Yanai and Sato, 1999)	Nucleus: 11–46-12.18 Mb, 7 chromosomes (Dujon et al., 2004) Mitochondrion: 29.5 Kb (Dujon et al., 2004)	2 genomes: CBS 767 and MTCC 234 (Dujon et al., 2004; Kumar et al., 2012)	Haploid (Breuer and Harms, 2006)	One (occasionally two) spores per ascus (Barnett et al., 2000). Zygotes described (Breuer and Harms, 2006)	–	Wild environments:ocean. Anthropic environments: cheese, grape.	–	–
*Pichia kluyveri*/*Hanseluna kluyveri*	Aromas (Anfang et al., 2009)	Mitochondrion: 43.1 Kb (CBS 7907)^1^.	–	Diploid (Starmer et al., 1992)	Four spores per ascus (Barnett et al., 2000). Zygotes described (Starmer et al., 1992)	–	Wild environments: fruit, insect. Anthropic environments: wine. (Starmer et al., 1992)	–	–
*Pichia kudriavzevii/Issatchenkia orientalis (Candida krusei)*	Under assessment (Clemente-Jimenez et al., 2004; Wang and Liu, 2013; Steensels and Verstrepen, 2014)	Nucleus: 10.18–12.94 Mb (Chan et al., 2012).	3 genomes:SD108, M12, NBRC 1279 (Chan et al., 2012)	Diploid	One or two spores per ascus (Barnett et al., 2000). Zygotes described^3^.	–	Wild environments: plant. Anthropic environments: wine, other traditional fermented beverages, food fermentation, dairy product. (Chan et al., 2012)	–	–
*Pichia membranifaciens (Candida valida)*	Esters production (Viana et al., 2008)	Nucleus: 11.58 Mb^2^, between 2 and 8 chromosomes (Naumov and Naumova, 2009)	1 genome^2^	–	One to four spores per ascus (Barnett et al., 2000).	–	Wild environments: plant. Anthropic environments: AF and food spoilage yeast.	–	–
*Pichia fermentans (Candida lambica)*	Aromas (Clemente-Jimenez et al., 2005)	Maybe 2 chromosomes (Miller et al., 1989).	–	–	Two to four spores per ascus (Barnett et al., 2000). Zygotes described^3^.	–	Wild environments: plant, water, soil. Anthropic environments:wine, brewery. Clinical environments.	–	–
*Pichia anomala/Hanseluna anomala (Candida pelliculosa)*	Aromas (Rojas et al., 2001; Domizio et al., 2011a,b); killer against Dekkera/Brettanomyces (Comitini et al., 2004)	Nucleus: 26.55 Mb, 6 chromosomes (Friel et al., 2005).	1 genome: NRRL Y-366^1^	Diploid	One to four spores per ascus (Barnett et al., 2000). Zygotes described^3^.	–	Wild environments:soil, water, plant, animal. Anthropic environments:wine, fermentation contaminant, ensilage (Kurtzman et al., 2011)	–	–
*Metschnikowia pulcherrima*/*Torulopsis pulcherrima (Candida pulcherrima)*	Aromas and esters production (Clemente-Jimenez et al., 2004; Parapouli et al., 2010; Zott et al., 2011; Sadoudi et al., 2012)	–	–	Diploid	One to two spores (Barnett et al., 2000).	–	Wild environments: plant. Anthropic environments: wine	–	–
*Zygosaccharomyces bailii*	Fructophily (Sutterlin, 2010)	Nucleus: 10.27–21.14 Mb, 5 to 13 chromosomes (Mira et al., 2014)	2 genomes: CLIB 213^T^ and ISA 1307 (NCBI^1^)	Haploid and diploid strains (Rodrigues et al., 2003)	One to four spores per ascus (Barnett et al., 2000).	–	Wild environments: fruit, tree. Anthropic environment: food spoilage	–	–

a Proportion of strains with heterozygous microsatellite loci

b Genetic diversity (0 means fully clonal population and 1 means fully diversified population)

*Web sites: NCBI^1^, http://www.ncbi.nlm.nih.gov/genome/; JGI^2^, http://genome.jgi.doe.gov/; UCDAVIS^3^, http://wineserver.ucdavis.edu/industry/enology/winemicro/wineyeast/diversity.html*.

## Basic genetic knowledge of wine yeasts

As a model organism, the genomic outline of *S. cerevisiae* is well-known: its genome size is around 12 Mb organized in 16 chromosomes, with a mitochondrial genome of 85 Kb (Table 1). The genome sequences of several hundreds of strains of various origins are available, and much more sequences are produced easily using NGS technology and subsequently assembled even by lab with moderate bioinformatics skills. The population genomics of *S. uvarum* has been improved recently with the sequencing of more than 50 strains of various origins (Almeida et al., 2014). The type strain CBS7001^T^ has a genome size of 11.5 Mb and 16 chromosomes (Cliften et al., 2003). By contrast, such basic knowledge (genome size, chromosome number, etc.) is available only for a small number of non-conventional wine species: *T. delbrueckii* has a genome of 9–11 Mb distributed on eight chromosomes; *L. thermotolerans* has a 10.4 Mb genome with eight chromosomes. Other wine yeast species usually have genome size ranging from 8 to 12 Mb, with chromosomes number unknown yet (*P. kluyveri, M. pulcherrima*, etc.). Moreover, there is still a lack of reference genome sequence for several non-conventional wine yeasts of interest like *S. bacillaris, P. fermentans*, etc., (Table 1). Disparities exist also for the mitochondrial genome, with full sequences available for some species like *L. thermotolerans* or *H. uvarum*, and partial sequences for other species (*C. stellata, P. membranifaciens*, etc.). Thus, although the genomic data of non-conventional wine yeast greatly increased this last decade, there is still a lot of work to achieve in this field.

## The life-cycle of wine yeasts

The life cycle of *Saccharomyces* wine species is well-known: both *S. cerevisiae* and *S. uvarum* are diploid species that divide asexually by mitosis. They are able to enter meiosis and form asci containing generally four haploid spores (tetrads). While haploid cells can undergo mitosis, the haploid level is generally transient and crosses between haploid spores of opposite mating types are readily observed, leading to diploid zygote formation. Moreover, haploid cells are usually able to switch mating type at mitosis (homothallism). The physical proximity between mother and daughter haploid cells of opposite mating type usually results in high level of inbreeding (Ruderfer et al., 2006; Cubillos et al., 2009; Warringer et al., 2011). Variations in this breeding system were described for *S. cerevisiae* like near-dioecy or higher level of outcrossing, but seemed quite rare and associated with environmental specificities (Knop, 2006; Al Safadi et al., 2010; Murphy and Zeyl, 2010).

By comparison, the precise life-cycle of most non-*Saccharomyces* yeasts is unknown yet. Sporulation was observed for most non-conventional yeast, albeit forming non-tetrad asci in many cases (*T. delbrueckii, D. hansenii, H. vinae*, etc., Table 1). No evidence of sporulation ability was recorded to date for *Starmerella/Candida* species. Data regarding the occurrence of sexual reproduction is usually scarce for most non-*Saccharomyces* yeasts, so classical genetic manipulations are impossible to date. To circumvent this limitation, both intra and inter specific hybridizations by protoplast fusion can be achieved as demonstrated in the past (Ball, 1984; Pina et al., 1986).

The basic ploidy level is also usually unresolved (Table 1): *T. delbrueckii* has been considered as a haploid species for a long time, but the detection of several strains harboring several loci with two alleles (26.4% of strains showing heterozygosity), its ability to sporulate and the presence of mating type genes is more congruent with a diploid status (Albertin et al., 2014a). Conversely, for *S. bacillaris*, the proportion of heterozygous strains was almost null (0.01%). This, combined with its inability to sporulate, is more consistent with an hypothesis of an haploid status (Masneuf-Pomarede et al., 2015) but has still to be formally demonstrated. Finally, despite its fully sequenced genome, the ploidy status of *L. thermotolerans* is controversial: haploid or diploid depending on the authors (Souciet et al., 2009; Freel et al., 2014). In conclusion, the biological life-cycle of many non-*Saccharomyces* yeasts remains to be elucidated.

## Ecology of wine yeast

Most wine yeasts can colonize several ecological niches, including wine-related environments like grape, must, winery equipment and premise (Table 1). Moreover, many of them can be isolated from other human-associated processes (brewery, bakery, dairy, bioethanol, distillery, etc.) and also from wild substrates (soil, insect, plant, etc.). Isolation from clinical specimens is rarely described yet possible (yeasts being opportunistic microorganisms), and most wine yeasts are Generally Recognized As Safe (GRAS). Dissemination and transfer between the different ecological reservoirs could be performed through insects (Parle and Di Menna, 1966; Stefanini et al., 2012; Palanca et al., 2013), but also through human activities like material exchanges, etc., (Goddard et al., 2010). Indeed, although most wine yeasts are described as ubiquitous from an ecological viewpoint, some species have a restricted substrate range. This is the case of *H. guillermondii* and *Starmerella* species for example, which are very rarely isolated from non-wine-related substrates (Masneuf-Pomarede et al., 2015). Thus, the study of most wine yeast should consider not only wine strains but also isolates from other technological processes and substrates in order to assess their biodiversity.

## Adaptation to winemaking environments and evolutionary mechanisms

Wine environments are particularly harsh and inconstant: winemaking is a seasonal practice, so that yeasts present at the surface of grape berries at harvest suddenly have to survive in grape must containing high sugar concentrations, usually with sulfur dioxide content. Moreover, from an ecological viewpoint, the ensuing alcoholic fermentation is a rapidly fluctuating ecosystem: within a few days, grape must is depleted of nitrogen nutrients, while ethanol concentration and temperature increase steadily thanks to *Saccharomyces* spp. metabolism, thus conferring a fitness advantage for *Saccharomyces* spp. over the other wine yeasts (Goddard, 2008; Salvadó et al., 2011). In addition, the range of temperature can be quite high, with either short-term variations (daily variations) or long-term evolution (seasonal variations). As a result, within wine yeast species, some strains show specific wine-adaptation (Steensels and Verstrepen, 2014) like sulphite resistance (Divol et al., 2012), ethanol tolerance (García-Ríos et al., 2014), low pH adaptation (Pretorius, 2000), temperature adaptation (Naumov et al., 2000), etc. The underlying adaptive mechanisms vary greatly from one species to another: in *S. cerevisiae*, molecular approaches identified allelic variations as molecular causes of adaptation to the winemaking process (Aa et al., 2006; Marullo et al., 2007; Ambroset et al., 2011; Salinas et al., 2012; Jara et al., 2014). At the chromosome level, translocations were shown to be responsible for adaptation to sulfite (Zimmer et al., 2014). Polyploidy and hybridization are also major evolutionary processes that probably triggered adaptation to wine environments (Borneman et al., 2012; Erny et al., 2012) and are currently explored for biotechnological application (Timberlake et al., 2011; Plech et al., 2014; Blein-Nicolas et al., 2015; da Silva et al., 2015). Large genomic introgressions were evidenced in *S. uvarum* strains associated with human-driven fermentations, suggesting a link between introgressions and domestication (Almeida et al., 2014). Various horizontal gene transfers were also evidenced for wine *S. cerevisiae* strains (Novo et al., 2009), and were shown to favor adaptation to the nitrogen-limited wine fermentation environment (Marsit et al., 2015). Other evolutionary mechanisms were described (Dujon et al., 2004; Barrio et al., 2006; Scannell et al., 2007), and it is highly probable that further investigations will allow the identification of additional adaptation processes in wine yeasts. In particular, it could be interesting to focus on transposon families and their possible implication in environmental adaptation (Zeyl, 2004; Liti et al., 2005; Sarilar et al., 2015), to explore the impact of mitochondrial genome variation regarding adaptation to wine environments and practices (Picazo et al., 2015; Wu et al., 2015) or to describe the landscape of gene duplication and prion involvement in fitness issues (Landry et al., 2006; Jarosz et al., 2014). However, to date, most of these data were obtained from *Saccharomyces* species and could now be obtained from non-*Saccharomyces* of interest.

## Population genetics of yeast species associated with winemaking

Within a given species, the colonization of different ecosystems can led to the evolutionary differentiation of the subpopulations, in relationship with their adaptation to environmental specificities. This is the case of *S. cerevisiae* species that shows genetic subgroups of wild and domestic strains associated with human activities like wine, bread, beer, sake, etc., (Fay and Benavides, 2005; Liti et al., 2009; Sicard and Legras, 2011; Almeida et al., 2015), that probably originated through multiple domestication events (Schacherer et al., 2009). In a recent study, Almeida et al. (2014) showed that *S. uvarum* was also divided in genetic subgroups, one of domestic strains used in both winemaking and cidermaking and associated with the northern hemisphere, while others subgroups were composed of wild isolates from South America and Australasia. The current hypothesis is that a Patagonian “wild” sub-population gave rise to the domestic subpopulation through a recent bottleneck (Almeida et al., 2014). Another wine species was recently described as domesticated: *T. delbrueckii* is also divided in genetic subgroups of wild and domestic strains (Albertin et al., 2014a). Moreover, the wine/grape-related group showed an increase ability to ferment sugar in oenological condition, confirming the occurrence of phenotypic domestication (Albertin et al., 2015). By contrast, no hint of domestication was recorded to date for *S. bacillaris* and *H. uvarum* whose genetic diversity is shaped by geographical localization and/or time variation (Masneuf-Pomarede et al., 2015; Albertin et al., 2016).

## Biodiversity in winemaking conditions

Several molecular methods were developed in order to perform intra-specific discrimination, like pulsed field electrophoresis, RAPD-PCR fingerprinting, tandem repeat-tRNA, Fourier transform infrared spectroscopy, RFLP, etc., (Barquet et al., 2012; Tofalo et al., 2013, 2014; Pfliegler et al., 2014; Grangeteau et al., 2015). However, these approaches do not allow the establishment of the genetic relationships within a given species and subsequent population genetics studies. An alternative is the use of microsatellite genotyping. It has been successfully applied to *S. cerevisiae* (Legras et al., 2005; Richards et al., 2009), *S. uvarum* (Masneuf-Pomarede et al., 2009), *T. delbrueckii* (Albertin et al., 2014a), *S. bacillaris* (Masneuf-Pomarede et al., 2015), *H. uvarum* (Albertin et al., 2016) as well as to the spoilage wine yeast *Brettanomyces bruxellensis* (Albertin et al., 2014c), and is currently developed for additional wine species like *Meyerozyma guilliermondii* (Wrent et al., 2015). In addition to population genetic clustering, microsatellites allow measuring the genetic diversity of a given species in specific conditions. In *S. cerevisiae*, the genetic diversity varied greatly, from 0 (fully clonal populations) to 1 (fully diversified population, Table 1). The precise impact of *S. cerevisiae* diversity (or absence of diversity) on wine quality is still debated/studied (Egli et al., 1998; Howell et al., 2006; King et al., 2008) and the direct link between microbial diversity and wine complexity should be considered with caution. *S. uvarum* and *T. delbrueckii* showed also a large range of diversity (0.35–1 and 0–0.62). By contrast, other species show systematic high diversity (>0.9 for *H. uvarum* or *S. bacillaris*), suggesting that they are not under selective pressure in winemaking environments (Masneuf-Pomarede et al., 2015; Albertin et al., 2016).

## Future challenges

Definite progresses in the genetics of non-conventional yeasts were made in the last decade. However, there is still a great lack of data compared to the conventional wine yeast *S. cerevisiae*. Such knowledge is nowadays within reach thanks to the NGS revolution (Solieri et al., 2013). NGS allows the development of genome-assisted approaches like whole genome sequencing and resequencing, transcriptome profiling, ChIP-sequencing to identify DNA-structure, etc., (Solieri et al., 2013). *De novo* sequencing is greatly needed as some wine species still lack of nuclear and mitochondrial reference genomes (*S. bacillaris, P. fermentans, M. pulcherrima*, etc.). However, *de novo* assembly is sometimes difficult to conduct due to high heterozygosity level or sequence repeat, and led to draft genome with high number of contigs or scaffolds. For example, *H. uvarum* DSM 2768 genome displays 335 contigs, *P. kudriavzevii* M12 has 621 scaffolds, and *P. anomala* NRRL Y-366 shows 1932 scaffolds. Thus, the first aim of non-conventional wine yeast studies should be the completion of robust genomic sequences. Then, additional genome sequencing could be performed: genome re-sequencing using NGS captures individual genotypes and allows population genetics and ecologic studies within species. Such comparative genomics approaches were successfully applied to *S. cerevisiae* (Liti et al., 2009) and *S. uvarum* (Almeida et al., 2014), and could now address non-*Saccharomyces* yeasts of technological interest. In addition to intraspecific genomics, comparative genomics between yeast species is particularly useful to understand genome evolution (Liti and Louis, 2005). The identification of specific metabolic pathways, gene duplications or functions between species may increase our appreciation of adaptation's mechanisms and their biotechnological interest (Blein-Nicolas et al., 2015). It has to be noted that several species genetically close to wine yeasts show no peculiar affinity with winemaking environment (Figure 1). This is the case of *S. paradoxus*: despite being the most closely related species to *S. cerevisiae, S. paradoxus* is essentially associated with wild environments and particularly trees (Sniegowski et al., 2002; Johnson et al., 2004). Comparative genomics of wine vs. non-wine yeast species could thus increase our knowledge of the common genomic requirement for grape/wine colonization, if any. Finally, NGS technologies have greatly improved genome-assisted approaches aiming at detecting genetic variants associated with phenotypes in *S. cerevisiae* (Ehrenreich et al., 2010). In particular, QTL-seq or genome-wide association studies (GWAS) could now be applied to non-conventional yeasts depending on whether classical breeding is possible (QTL-seq) or not (GWAS). These fields are blank pages waiting to be filled in the next future of oenology microbial research.

The use of mixed-cultures, combining both non-conventional yeasts and one *Saccharomyces* species able to complete AF, is increasing in winemaking. Thus, another challenge lies in understanding yeast-yeast interactions and their underlying mechanisms (Ciani et al., 2010; Ciani and Comitini, 2015). Indeed, several types of yeast-yeast interactions have been described in enological conditions: competition for nutriments, release of toxic compounds (Fleet, 2003), and even “quorum-sensing” like mechanisms (Nissen and Arneborg, 2003; Nissen et al., 2003; Renault et al., 2013). Understanding these complex interactions is of first importance as the combination of some yeast strains seems condemned to failure: for example, cell-cell contact was recently shown to be involved in the death of strains of *T. delbrueckii* and *L. thermotolerans* during mixed-culture alcoholic fermentation with *S. cerevisiae* (Renault et al., 2013; Kemsawasd et al., 2015). In some cases, yeast death was associated with the release of metabolites or killer toxin (Pérez-Nevado et al., 2006; Albergaria et al., 2010; Branco et al., 2015; Ramírez et al., 2015). The precise impact of such interactions regarding wine quality and aromas is still unclear (Ciani et al., 2006), but will have to be considered to control and optimize complex mixed oenological fermentation.

Finally, in addition to NGS-assisted approaches and interactions studies, another prospect in the field of non-conventional wine yeast lies in classical genetic approaches: indeed, one of the limits of the previously detailed approaches is their low ability in elucidating the basic life-cycle of wine yeasts, particularly regarding the occurrence and control of sexual reproduction. Still, classical breeding is one of the key issues for genetic improvement of industrial strains of *S. cerevisiae* (Pretorius, 2000; Giudici et al., 2005; Marullo et al., 2006; Steensels et al., 2014) and represents a technological barrier that must be overcome for actual improvement of non-*Saccharomyces* wine yeasts. There is an important need for traditional sporulation assays, spore microdissection attempts, subsequent segregant analyses, breeding assays, etc. In addition, genetic transformation of non-conventional wine yeasts would be a welcomed tool for subsequent functional studies (Pacheco et al., 2009; Roberts and Oliver, 2011). These classical approaches are time-consuming and necessitate traditional yeast-manipulation know-how, sometimes viewed as old-fashioned and therefore neglected. However, these old approaches are essential for our future understanding of the genetics of non-conventional wine yeast, and are complementary to the more *en vogue* NGS-assisted approaches.

## Funding

This work was funded by the European Union's Seventh Framework Programme (FP7/2007–2013) under grant agreement n° 315065 WILDWINE project: “Multi-strain indigenous Yeast and Bacterial starters for “Wild-ferment” wine production.”

### Conflict of interest statement

The authors declare that the research was conducted in the absence of any commercial or financial relationships that could be construed as a potential conflict of interest.
